# Adjuvant and neoadjuvant therapies in HCC: Time to go perioperative?

**DOI:** 10.1016/j.isci.2026.117084

**Published:** 2026-08-10

**Authors:** Spyridon Pantzios, Georgios Germanidis, Ioannis Elefsiniotis

**Affiliations:** 1 Academic Department of Internal Medicine – Hepatogastroenterology Unit, General Oncology Hospital of Kifisia “Agioi Anargyroi”, National and Kapodistrian University of Athens, Athens, Greece; 2 Division of Gastroenterology and Hepatology, First Department of Internal Medicine, AHEPA University Hospital, Aristotle University of Thessaloniki, Thessaloniki, Greece; 3 Basic and Translational Research Unit, Special Unit for Biomedical Research and Education, School of Medicine, Faculty of Health Sciences, Aristotle University of Thessaloniki, Thessaloniki, Greece

**Keywords:** HCC, adjuvant, neoadjuvant, perioperative, immunotherapy

## Abstract

Hepatocellular carcinoma (HCC) remains a leading cause of cancer-related mortality worldwide, with high recurrence rates despite curative-intent therapies. Immune checkpoint inhibitors (ICIs) have improved outcomes in advanced disease and are now being explored in earlier stages. However, adjuvant approaches have shown limited and inconsistent benefit across phase III trials. In contrast, neoadjuvant strategies have demonstrated promising activity, likely due to enhanced immune priming in the presence of an intact tumor, leading to improved pathological responses and potential tumor downstaging. Emerging evidence suggests that perioperative approaches combining neoadjuvant and adjuvant immunotherapy may provide a more comprehensive strategy by targeting both macroscopic and microscopic disease. Early phase III data indicate improved oncological outcomes and possible conversion to resectability in selected patients. Nonetheless, challenges remain, including patient selection, optimal sequencing, toxicity, and lack of predictive biomarkers. This review summarizes current evidence and future perspectives on adjuvant, neoadjuvant and perioperative systemic therapies in HCC.

## Graphical abstract

**Figure:**
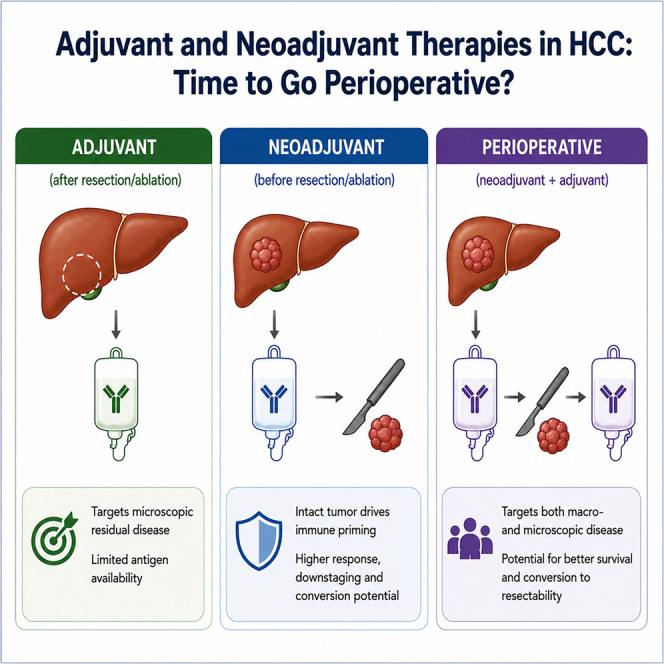

## Teaser

Oncology; Therapeutics

## Introduction

Liver cancer represents one of the most prevalent and lethal malignancies of the gastrointestinal tract, ranking as the sixth most commonly diagnosed cancer and the third leading cause of cancer-related mortality worldwide.^1^ Hepatocellular carcinoma (HCC) accounts for approximately 90% of primary liver cancer cases.^2^ The prognosis of patients with HCC is closely associated with intrahepatic tumor burden, the presence of extrahepatic spread, macrovascular invasion and liver disease severity.^3^ Notably, a substantial proportion of patients are diagnosed at advanced stages of disease, thereby limiting the applicability of curative-intent therapies and contributing to poor overall survival (OS) outcomes.^4^

Over the past five years, the introduction of immunotherapy-based regimens has significantly reshaped the therapeutic landscape of advanced HCC.^5^ These approaches have demonstrated meaningful improvements in OS, alongside objective response rates (ORRs) approaching 30–35% in selected patient populations.^6^ The landmark IMbrave150 trial established the combination of atezolizumab and bevacizumab as a superior first-line treatment compared to sorafenib, highlighting the critical role of immune modulation and angiogenesis inhibition in advanced HCC management.^7^ Subsequently, the HIMALAYA trial demonstrated that dual immune checkpoint blockade with tremelimumab (anti-CTLA4) and durvalumab (anti-PDL1) provides a survival benefit over sorafenib, thereby introducing the STRIDE regimen as an alternative first-line therapeutic option.^8^ Importantly, long-term follow-up data have shown that a subset of these patients (approximately 20%) may achieve durable responses, with survival extending up to five years.^9^ More recently, the CheckMate-9DW study evaluating nivolumab in combination with ipilimumab further expanded the spectrum of effective immunotherapeutic strategies in advanced HCC.^10^

The clinical success of immune checkpoint inhibitors (ICIs), either as monotherapy or in combination with anti-VEGF agents, in advanced-stage disease has prompted increasing interest in their evaluation in earlier stages of HCC.^11^ Indeed, over the past three years, a growing body of evidence has emerged investigating these agents not only in intermediate-stage disease but also in early-stage settings, where curative-intent treatments such as surgical resection, local ablation or liver transplantation remain feasible and potentially offer disease-free long-term survival.^12^

In this context, two therapeutic strategies widely implemented in other solid malignancies—namely adjuvant and neoadjuvant systemic therapy—are gaining attention in HCC management.^13^ These approaches could be integrated as adjuncts to curative modalities, with the aim of reducing recurrence risk, downstaging tumors and improving long-term outcomes. Despite their promising rationale, the use of adjuvant and neoadjuvant systemic therapies is not currently endorsed by international clinical guidelines for HCC.^14^^,^^15^ Nevertheless, ongoing clinical trials and emerging data suggest that these strategies may become incorporated into treatment algorithms for selected patient populations in the near future.^16^

The aim of this review is to summarize the available evidence regarding neoadjuvant, adjuvant, and perioperative systemic therapies in HCC, including combination strategies, and to critically discuss their potential clinical applications, limitations and future perspectives within the evolving therapeutic landscape of HCC.

## Rationale for using adjuvant immunotherapies in HCC

In the field of oncology, adjuvant treatment strategies have been widely implemented across a broad spectrum of solid tumors.^17^ In the context of HCC, this concept is particularly relevant. Despite apparently curative treatments, there remains a substantial likelihood that microscopic neoplastic foci persist within the liver parenchyma.^18^ Consequently, the rationale for adjuvant therapy in HCC lies in the eradication of these occult lesions, thereby decreasing early postoperative recurrence rates and potentially improving long-term survival outcomes.^19^

The risk of recurrence following radical treatment for HCC is influenced by a variety of factors that can be broadly categorized into tumor-related, patient-related and treatment-related parameters. Tumor-related characteristics include biological aggressiveness, histological grade, presence of microvascular invasion, existence of satellite or micronodular lesions, elevated serum alpha-fetoprotein (AFP) levels and tumor size – particularly lesions exceeding 5 cm in diameter.^20^ These features are consistently associated with a higher likelihood of early recurrence and poorer prognosis. Patient-related factors also play a crucial role in determining recurrence risk and OS. These include tumor stage at diagnosis and the presence of underlying liver disease, most notably cirrhosis.^21^ Cirrhosis not only limits hepatic functional reserve, but also constitutes a pro-oncogenic environment that predisposes to the development of new primary tumors.^22^ Indeed, recurrence in HCC may result not only from residual microscopic disease but also from *de novo* carcinogenesis, particularly in cirrhotic livers, further complicating postoperative management.^23^ Additional risk factors have been identified, including the presence of multiple tumors at diagnosis, the requirement for excessive intraoperative blood transfusion and male gender.^24^

Moreover, HCC presents unique clinical challenges due to its frequent association with chronic liver disease. This coexistence may restrict the extent of liver resection that can be safely performed, potentially leading to suboptimal or subtherapeutic resections and, consequently, an increased risk of recurrence.^25^ Another important issue in the management of HCC is the lack of universal consensus regarding resectability criteria. A substantial “gray zone” exists between clearly resectable and definitively unresectable disease, encompassing patients with marginally resectable tumors.^26^ This ambiguity contributes to variability in clinical decision-making and patient selection for surgical intervention. Furthermore, in certain regions, there is a tendency to extend surgical indications to patients with more advanced disease, including those with intermediate-stage HCC or even selected cases with segmental macrovascular invasion.^27^ While this approach may offer potential survival benefits in highly specialized centers, it is inevitably associated with increased postoperative recurrence rates. Despite advances in surgical techniques, perioperative care and patient selection, recurrence remains a major limitation in the management of HCC. Even in high-volume centers with considerable expertise, the 5-year recurrence rate following curative resection ranges from 30% to 40%.^28^ As a result, the use of adjuvant systemic treatment is a very attractive option, especially in high-recurrence-risk patients.

## Pros, challenges and limitations of adjuvant therapies

The administration of adjuvant therapy in patients with HCC offers several theoretical and practical advantages. One of the main benefits lies in the ability to incorporate detailed pathological and histological information obtained following curative-intent treatments, such as liver resection.^29^ This allows clinicians to better characterize tumor biology and identify established prognostic factors associated with recurrence risk, including microvascular invasion, tumor differentiation and microscopic tumor burden.^30^ An additional advantage of adjuvant therapy is that it does not interfere with the timing or feasibility of the scheduled primary curative intervention.^31^ In contrast to neoadjuvant approaches, where concerns may arise regarding treatment-related toxicity or disease progression prior to surgery, adjuvant therapies are administered after definitive tumor removal and therefore function as a complementary component of the overall treatment strategy. This ensures that the primary objective of achieving complete tumor eradication is not compromised or delayed.

Despite these advantages, several important challenges and limitations remain, which currently hinder the widespread implementation of adjuvant systemic therapies in HCC. One of the most critical issues is the identification of the appropriate patient population. Although multiple studies have attempted to define “high-risk” disease, there is still no universally accepted set of criteria that can reliably predict which patients are most likely to benefit from adjuvant treatment.^19^^,^^32^ The heterogeneity of HCC, both in terms of tumor biology and underlying liver disease, further complicates patient stratification and limits the generalizability of clinical trial results.^33^

Another major challenge relates to the optimal timing of adjuvant therapy initiation following curative treatment.^34^ Most clinical trials have adopted a treatment window of approximately 4–8 weeks after surgery or ablation; however, this time frame is largely empirical and not supported by robust biological or clinical evidence.^35^^,^^36^^,^^37^^,^^38^ In addition, the timing of therapy initiation may need to be individualized depending on the type of systemic regimen used. For instance, patients receiving anti-VEGF-based therapies may require a longer delay prior to treatment initiation due to the increased risk of bleeding and impaired wound healing associated with these agents.^39^^,^^40^ The optimal duration of adjuvant therapy also remains undefined. While many studies have employed a treatment duration of approximately 6–12 months, this approach is largely extrapolated from other malignancies and lacks specific validation in HCC.^41^^,^^42^ Prolonged exposure to systemic therapy raises additional concerns regarding cumulative toxicity, patient compliance and cost-effectiveness, all of which must be carefully balanced against potential clinical benefit.^43^

A further limitation of adjuvant immunotherapy is the absence of reliable methods to assess treatment response in the context of microscopic residual disease.^44^ Unlike advanced disease settings, where radiological imaging can provide measurable endpoints, the adjuvant setting lacks robust tools to dynamically monitor therapeutic efficacy. Emerging approaches, such as liquid biopsy techniques—including the analysis of circulating tumor cells (CTCs) and circulating tumor DNA (ctDNA)—offer a promising avenue for real-time assessment of minimal residual disease and tumor dynamics.^45^ However, at present, there is insufficient evidence to support the routine use of these biomarkers in clinical practice and their correlation with intrahepatic tumor biology remains to be fully validated.^46^ One of the most clinically relevant and unresolved challenges is the early identification of patients who do not respond to adjuvant therapy, ideally before radiological evidence of recurrence becomes apparent.^47^ The inability to detect treatment failure at an early stage may result in prolonged exposure to an ineffective therapy, potentially delaying the initiation of alternative and more appropriate subsequent treatment strategies.

On the other hand, increasing attention has recently been directed toward the role of tertiary lymphoid structures (TLSs) within the HCC tumor microenvironment. It is well established that a highly inflamed tumor microenvironment, characterized by abundant infiltration of cytotoxic CD8^+^ T lymphocytes, is associated with improved responses to ICIs and superior survival outcomes across several solid malignancies.^48^ TLSs are ectopic lymphoid aggregates that develop in tissues under conditions of chronic inflammation and consist of organized immune cell niches containing T cells, B cells, dendritic cells and memory lymphocytes, thereby serving as sites of local antigen presentation and adaptive immune activation.^49^ Although earlier studies suggested that the presence of TLSs might be associated with unfavorable outcomes in HCC, more recent evidence has challenged this concept.^50^ In a cohort of 273 patients undergoing curative resection, intratumoral TLS were associated with a significantly lower risk of early recurrence, supporting their role as a marker of an active antitumor immune microenvironment.^51^ More importantly, a recent study demonstrated that patients with resected HCC who received adjuvant anti-PD1 therapy derived a significantly greater recurrence-free survival (RFS) benefit when TLSs were present within the tumor, whereas patients lacking TLSs did not appear to benefit from adjuvant immunotherapy.^52^ These findings suggest that TLSs may represent one of the first clinically relevant predictive biomarkers for patient selection in the adjuvant setting. Together with other emerging tissue-based biomarkers, including the characterization of tumor-infiltrating lymphocytes, TLSs may facilitate the development of biomarker-driven postoperative treatment strategies and future clinical trials by identifying the subgroup of patients most likely to benefit from adjuvant immunotherapy. Such an approach could partly explain the disappointing results observed in the unselected populations enrolled in trials such as IMbrave050 and KEYNOTE-937 and represents an important step toward precision medicine in early-stage HCC. Furthermore, the presence of microvascular invasion in the resected tumor, might indicate a subgroup of patients that can demonstrate better efficacy results with adjuvant immunotherapy, as presented by a recent multicenter phase II study from China with anti-PD1 immunotherapy in patients with resected HCC with microvascular invasion.^53^ Ultimately, the biological information obtained from the resected tumor should be fully exploited to guide postoperative therapeutic decisions rather than relying solely on conventional clinicopathological risk factors.

Finally, the use of ICIs in the adjuvant setting raises important concerns regarding subsequent treatment options in the event of disease recurrence. Patients who experience relapse during or shortly after completing adjuvant immunotherapy may have already developed primary or acquired resistance to these agents through a variety of mechanisms.^54^ Consequently, clinicians may preferentially consider alternative second-line systemic therapies, most commonly tyrosine kinase inhibitors (TKIs), which generally provide more modest response rates and survival benefits than immunotherapy-based regimens in advanced HCC.^55^ Nevertheless, ICI rechallenge has recently emerged as a promising therapeutic strategy. A large multicenter retrospective study from China including 272 patients demonstrated ORRs of approximately 25% following ICI rechallenge, with a favorable and manageable safety profile, particularly when alternative combination strategies were employed rather than repeating the same immunotherapeutic regimen.^56^ Similar efficacy has also been reported in a large European retrospective study, in which second-line ICI-based therapy achieved comparable ORRs and a median time-to-progression of approximately five months, similar to that observed during first-line immunotherapy.^57^ Importantly, both studies demonstrated that the safety profile of ICI rechallenge was consistent with that observed during initial immunotherapy and did not reveal any unexpected toxicities. Collectively, these findings suggest that prior exposure to immunotherapy should not automatically preclude subsequent ICI-based treatment in carefully selected patients.^58^ However, prospective studies are still required to better define the optimal candidates, treatment sequencing and mechanisms underlying successful immune rechallenge in HCC.

## Current data from clinical trials on adjuvant therapies in HCC

The integration of adjuvant systemic therapies in HCC has been investigated for over a decade, with the primary aim of reducing the high rates of postoperative recurrence following curative-intent treatments.^59^ The first large randomized controlled trial in this setting was the STORM trial, which evaluated the use of a TKI, sorafenib, as an adjuvant therapy compared to placebo in patients with HCC at high risk of recurrence after liver resection or local ablation.^60^ High-risk disease in this study was defined by the presence of adverse pathological and clinical features, including microvascular invasion, satellite nodules, poor tumor differentiation and diffuse multinodular disease. Despite the strong rationale for targeting residual microscopic disease, the trial failed to meet its primary endpoint, as no significant difference in RFS was observed between the two groups (33.3 months in the sorafenib arm versus 33.7 months in the placebo arm; *p* = 0.26). Similarly, no improvement in OS was demonstrated. These negative results represented a major setback in the field and led to a relative stagnation of research efforts in adjuvant HCC for nearly a decade.

Renewed interest in adjuvant strategies emerged with the development of ICIs, culminating in the IMbrave050 trial, the first phase III study to evaluate immunotherapy in the adjuvant setting for HCC.^38^ This trial compared the combination of atezolizumab and bevacizumab with active surveillance in patients who had undergone liver resection or ablation and were considered at high risk of recurrence. High-risk features included multinodular disease, microvascular invasion and the presence of satellite lesions, even in tumors with relatively low histological grade. In the first interim analysis at 12 months, the study reported a statistically significant improvement in RFS with the combination therapy. However, this initial benefit was not sustained in subsequent analyses at 24 and 36 months. Ultimately, no statistically significant difference in RFS was observed between the two arms (33.2 months in the atezolizumab-bevacizumab group versus 36 months in the active surveillance group). Importantly, subgroup analyses demonstrated no significant benefit from adjuvant therapy even when patients were stratified according to tumor burden, including those within and beyond the up-to-7 criteria.^61^ These findings remained consistent despite the application of alternative statistical methodologies that accounted for non-proportional hazards, a known limitation of the original analysis given that the log rank test may not have been optimal in this context.^62^ An additional factor that should be reported is that half of the patients in the active treatment arm discontinued therapy due to grade 3 or higher adverse events. This high discontinuation rate raises important considerations regarding the feasibility of prolonged immunotherapy in the postoperative setting.

More recently, the results of the KEYNOTE-937 trial were presented.^37^ This phase III study evaluated pembrolizumab versus placebo as adjuvant therapy in patients with HCC who had undergone resection or ablation and were classified as having intermediate, high or very high risk of recurrence. Although detailed data regarding microvascular invasion status have not yet been fully reported, the study population encompassed a broad spectrum of intrahepatic disease, ranging from solitary tumors to multifocal disease with varying tumor sizes. Similar to the findings of IMbrave050, pembrolizumab failed to demonstrate a statistically significant improvement in RFS compared to placebo (46.7 months in the pembrolizumab arm versus 45.5 months in the placebo arm; *p* = 0.719). Furthermore, in this study, median OS was not reached in either arm due to immature data. Additionally, distant metastasis-free survival, another exploratory endpoint, also remained immature at the time of analysis. Regarding safety, serious adverse events were reported in 14% of patients receiving pembrolizumab, while 10% of patients discontinued treatment due to toxicity.

The failure of these systemic agents in the adjuvant setting, despite their well-established efficacy in advanced HCC, raises important questions regarding the biological differences between early and late-stage disease.^63^ One potential explanation lies in the distinct tumor microenvironment and antigenic landscape of early-stage HCC.^64^ Advanced tumors are typically more biologically aggressive, genetically complex and heterogeneous, with a higher tumor mutational burden and increased neoantigen load, all of which may contribute to enhanced immunogenicity and improved responsiveness to immunotherapy.^65^ In contrast, early-stage tumors may consist of more biologically indolent or “dormant” tumor cell populations, characterized by lower antigenicity and reduced capacity to effectively prime the immune system.^66^ In the absence of a sufficient antigenic stimulus, the ability of ICIs to generate a robust and sustained anti-tumor immune response may be significantly diminished, potentially explaining the lack of efficacy observed in the adjuvant setting.^67^

Conversely, a multicenter randomized phase II study conducted in China evaluated adjuvant sintilimab, a PD-1 inhibitor, in patients with resected HCC presenting with microvascular invasion, a well-established predictor of early postoperative recurrence.^53^ Patients received either six months of adjuvant sintilimab or active surveillance. Among the 198 enrolled patients, adjuvant sintilimab significantly improved RFS compared to active surveillance (median RFS of 27.7 vs. 15.5 months; *p* = 0.002, respectively). OS data remained immature, with the median OS not reached in either group at the time of analysis. Interestingly, the greatest benefit was observed among patients with baseline AFP levels >20 ng/mL, suggesting that specific biological subgroups may derive greater benefit from adjuvant immunotherapy. Treatment-related adverse events occurred in approximately 85% of patients receiving sintilimab, although most were grade 1–2 and consistent with the established safety profile of the drug. Importantly, only 8% of patients discontinued treatment because of treatment-related toxicity, supporting the overall feasibility of this approach. Unlike the negative findings of IMbrave050 and KEYNOTE-937, this study demonstrated a clear improvement in RFS with adjuvant immune checkpoint inhibition. Several factors may account for these results. First, this was a phase II trial that enrolled a highly selected population consisting exclusively of patients with microvascular invasion, thereby enriching the cohort for individuals at particularly high risk of early recurrence and potentially more likely to benefit from adjuvant therapy. In contrast, IMbrave050 and KEYNOTE-937 were phase III trials that included broader and biologically more heterogeneous high-risk populations. Second, the study was conducted exclusively in centers in China, where the underlying etiological profile of HCC and tumor biology differ from Western populations, potentially limiting the generalizability of the findings. Consequently, although these results are encouraging, validation in large international phase III trials will be necessary before adjuvant sintilimab can be incorporated into routine clinical practice.

Finally, several additional adjuvant systemic regimens are currently under investigation and are summarized in Table 1. The results of these trials are eagerly awaited and are expected to provide further insight into the potential role of adjuvant therapy in HCC, ultimately contributing to a more comprehensive understanding of the use of these systemic treatments in earlier stages of disease.

**Table 1:** Major ongoing clinical trials in HCC adjuvant immunotherapy

Trial	Test arm	Comparator	Patient population	Primary endpoint	NCT no.
CheckMate 9DX	nivolumab	placebo	high-risk of recurrence HCC after radical resection/ablation	RFS	NCT03383458
Jupiter 04	toripalimab	placebo	high-risk of recurrence HCC after radical resection	RFS	NCT03859128
EMERALD-2	durvalumab + bevacizumab or durvalumab monotherapy	placebo	high-risk of recurrence HCC after radical resection/ablation	RFS	NCT03847428

NCT: national clinical trial; HCC: hepatocellular carcinoma; RFS: recurrence-free survival.

## Rationale for using neoadjuvant immunotherapies in HCC

In contrast to adjuvant strategies, neoadjuvant treatment is administered to patients who are not considered optimal candidates for immediate curative-intent interventions due to a more extensive tumor burden or borderline resectability.^68^ In this setting, the upfront use of systemic therapy aims not only to reduce tumor burden and facilitate subsequent radical procedures, but also to modulate tumor biology in a manner that may improve long-term oncologic outcomes. This therapeutic paradigm has been widely adopted across multiple solid malignancies, where neoadjuvant approaches have demonstrated benefits in terms of tumor downstaging, early eradication of micrometastatic disease and improved survival.^69^^,^^70^^,^^71^^,^^72^ In HCC, a disease characterized by both complex tumor biology and an immunologically distinct microenvironment, this strategy has increasingly been proposed as a potentially valuable addition to the therapeutic armamentarium.^73^

Recent translational and clinical studies provide strong support for the biological rationale underlying neoadjuvant immunotherapy importance in HCC in order to further enhance immune-mediated antitumor activity.^74^ A recent study by Ho et al. demonstrated that the combination of the TKI cabozantinib with the anti-PD1 molecule nivolumab in the neoadjuvant setting not only enabled conversion of locally advanced tumors to resectable disease, but was also associated with enhanced intratumoral immune infiltration in responders compared to non-responders, as explained by enrichment in T-cells, B-cells and plasma cells arranged in TLSs and enhanced activation of cytotoxic T cell responses, as can be noted from the elevated granzyme expression in responders. Importantly, responders exhibited increased immune cell infiltration both at baseline and following treatment, suggesting that the presence of an intact tumor microenvironment facilitates effective immune priming.^75^ Similarly, the more recent study by Magen et al. confirmed that neoadjuvant anti-PD1 immunotherapy induces the proliferation of T-helper and non-exhausted T-cytotoxic cells in responders, while an immune-exhausted CD8^+^ T cell population was the dominant population in non-responders. Furthermore, the presence of dendritic and CD4^+^ T-cells in niches within the tumor microenvironment could be associated with the differentiation of CD8^+^ T-cells upon receipt of neoadjuvant immunotherapy.^76^ These findings support the concept that preoperative administration of immunotherapy maximizes antigen exposure and immune activation, thereby generating a more robust and systemic anti-tumor response compared to adjuvant approaches.

**Figure 1:**
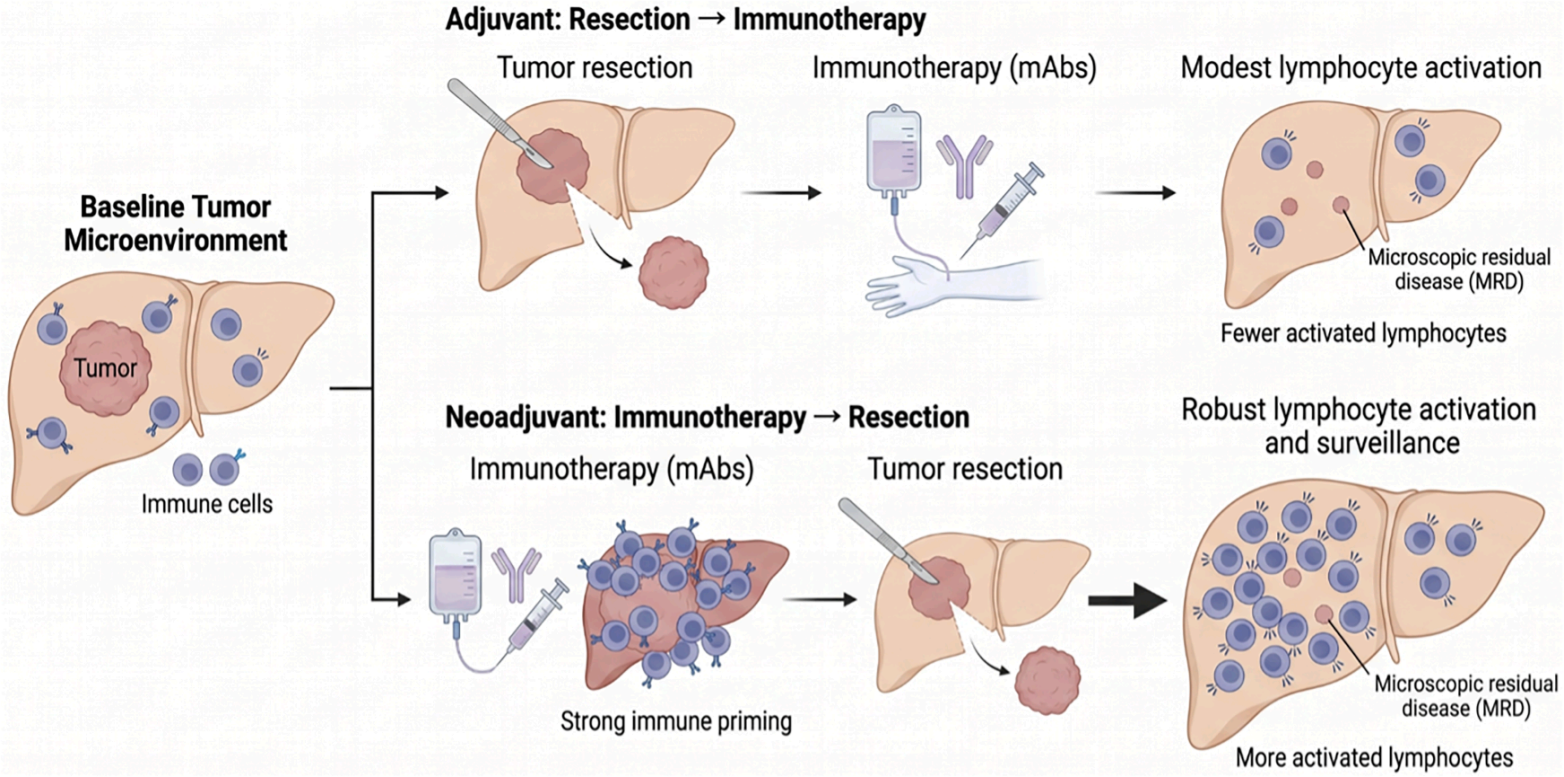
Conceptual comparison of adjuvant versus neoadjuvant immunotherapy in hepatocellular carcinoma (HCC) Schematic representation of the immunological and therapeutic differences between adjuvant and neoadjuvant strategies. In the adjuvant approach (upper image), tumor resection is performed prior to systemic therapy, resulting in limited antigen availability postoperatively and modest immune activation in the presence of microscopic residual disease (MRD). This may lead to suboptimal lymphocyte priming and reduced immune surveillance. In contrast, the neoadjuvant approach (lower image) involves administration of immunotherapy in the presence of an intact tumor, allowing for enhanced antigen presentation and robust immune priming. This results in increased activation and expansion of tumor-specific lymphocytes prior to surgical resection, potentially improving postoperative immune surveillance and control of MRD. mAbs: monoclonal antibodies; MRD: microscopic residual disease.

As noted, the biological rationale supporting neoadjuvant immunotherapy in HCC is primarily based on the concept of enhanced immune priming in the presence of an intact tumor.^77^ Prior to surgical resection or local ablation, the tumor serves as a rich and continuous source of tumor-associated antigens, enabling effective antigen presentation and subsequent activation of tumor-specific T cells.^78^ This process facilitates the recognition of a broad repertoire of tumor epitopes, thereby lowering the threshold for immune activation and promoting the expansion of a more diverse and robust anti-tumor immune response.^79^ Following this initial phase of immune activation, the execution of a radical procedure may occur in a host environment already primed for tumor control, characterized by increased infiltration and activation of cytotoxic lymphocytes and other effector immune cells.^80^ In contrast, the administration of adjuvant therapy after tumor removal is inherently limited by the absence of a substantial antigenic stimulus, as only minimal or microscopic residual disease may remain, as depicted in Figure 1. Consequently, neoadjuvant administration of ICIs may lead to more potent and sustained immune-mediated tumor control.^11^ Indeed, emerging data suggest that such preoperative immune priming may translate into higher rates of pathological response, which in turn have been associated with improved postoperative outcomes, even among patients presenting with more advanced disease features.^81^

The potential integration of neoadjuvant strategies is particularly relevant for patients who fall just beyond the conventional eligibility criteria for curative treatments.^82^ In these borderline cases, neoadjuvant systemic therapy may facilitate tumor downstaging, thereby enabling access to surgical resection or transplantation in selected patients who would otherwise be excluded from these options.^83^ Furthermore, in cases where more aggressive surgical approaches are adopted, the broader application of resection in intermediate or even selected advanced-stage HCC, provides a valuable clinical context in which the efficacy of neoadjuvant systemic therapies can be further explored and validated.^84^

Despite this compelling biological and clinical rationale, the current evidence supporting neoadjuvant strategies in HCC remains limited. Most available data are derived from early-phase, single-arm studies with relatively small patient populations, heterogeneous inclusion criteria and variable endpoints. At the same time, the persistently high rates of recurrence following curative-intent treatments underscore a substantial unmet clinical need in HCC management.^85^ This reality highlights the importance of optimizing perioperative therapeutic strategies, including both neoadjuvant and adjuvant approaches, with the ultimate goal of reducing recurrence risk and improving long-term survival.^86^

## Pros, challenges and limitations of neoadjuvant therapies

**Figure 2:**
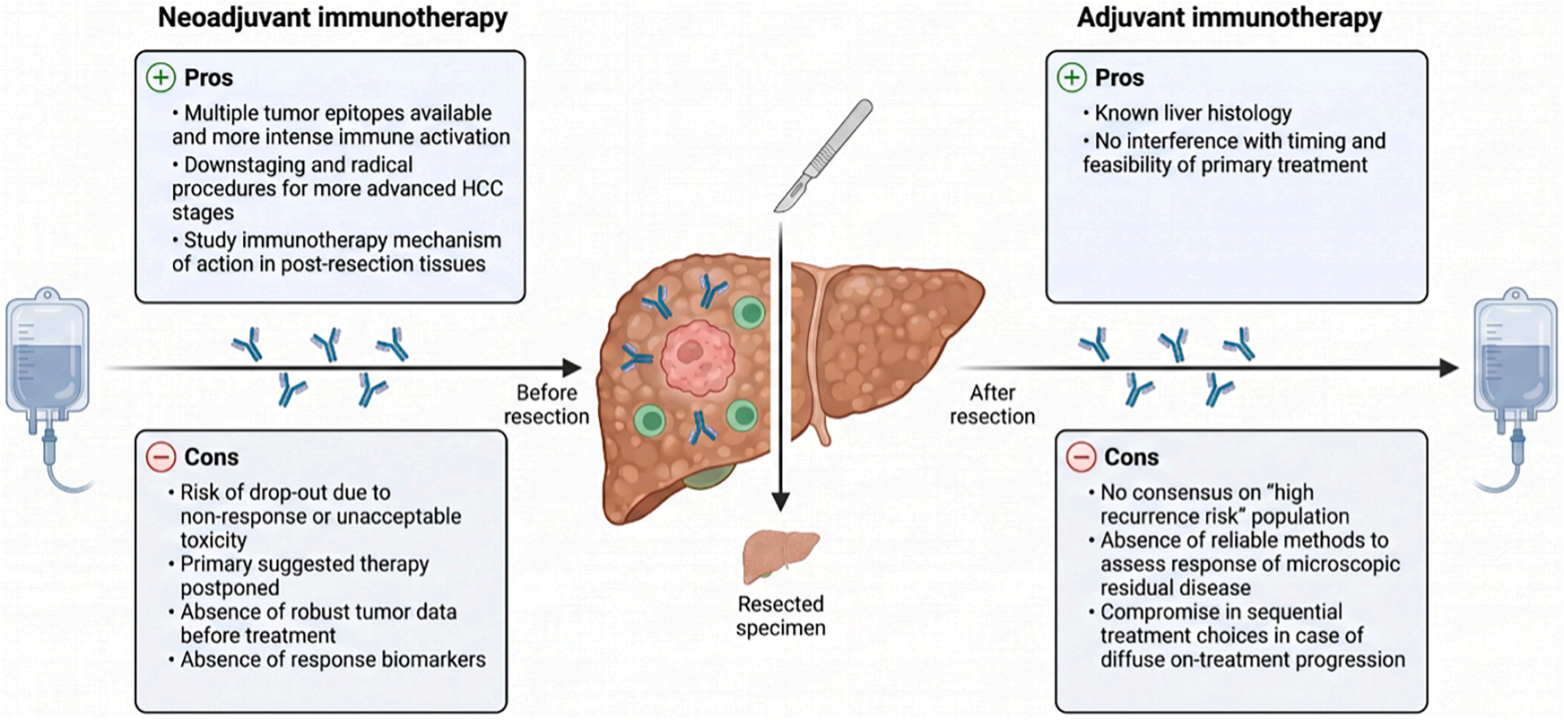
Comparison of neoadjuvant versus adjuvant immunotherapy strategies in hepatocellular carcinoma (HCC) This schematic illustrates the key differences between neoadjuvant and adjuvant immunotherapy approaches in HCC. Neoadjuvant immunotherapy, administered prior to tumor resection, allows for enhanced immune priming due to the presence of an intact tumor rich in antigenic epitopes, potentially leading to stronger systemic anti-tumor responses and facilitating downstaging. However, it carries risks such as treatment-related toxicity, potential delay of definitive therapy and lack of established predictive biomarkers. In contrast, adjuvant immunotherapy is administered after surgical resection, benefiting from detailed histopathological assessment and preserving the timing of curative treatment. Nevertheless, its efficacy may be limited by reduced antigenic stimulation from minimal residual disease, as well as challenges in patient selection and monitoring treatment response.

Neoadjuvant systemic therapy also offers several important theoretical and clinical advantages in the management of HCC, as presented in Figure 2. One of its most significant benefits is the potential to convert patients who are initially ineligible for curative-intent treatments into candidates for radical therapeutic approaches.^87^ By reducing tumor burden and modifying tumor biology, neoadjuvant strategies may facilitate downstaging and expand the pool of patients eligible for definitive treatment. In addition, the administration of systemic therapy in the presence of an intact tumor may result in a more robust immune activation compared to adjuvant approaches, as the tumor provides a continuous source of antigenic stimulation, thereby enhancing immune priming and promoting a broader anti-tumor response.^77^^,^^88^

Despite these advantages, several important limitations must be considered. Unlike adjuvant therapy, which benefits from the knowledge of a detailed histopathological assessment of the resected tumor, neoadjuvant treatment is often initiated without comprehensive characterization of tumor biology. In many cases, diagnosis is established radiologically; however, in selected patients, a tissue biopsy may be required according to contemporary clinical guidelines.^15^ Needle biopsy, while generally safe, has inherent limitations, including the risk of sampling error and underestimation of tumor heterogeneity, particularly when necrotic or non-representative tissue is obtained.^89^ Furthermore, although rare in experienced centers, there remains a risk of tumor seeding along the biopsy tract of approximately 2.7%, which may be of particular concern in less experienced centers.^90^

A major challenge associated with neoadjuvant therapy is the uncertainty regarding treatment response. Although emerging data suggest that the presence of intratumoral TLSs is associated with the administration of neoadjuvant therapy, it is generally not clear if patients responding to treatment are patients with the presence of TLSs *per se* or if TLSs appearance is a result of the administration of neoadjuvant immunotherapy itself.^91^^,^^92^^,^^93^ At present, there are no widely accepted reliable biomarkers to predict response, even in the advanced HCC setting.^94^ Consequently, the use of neoadjuvant treatment inherently involves a degree of clinical risk. Patients who fail to respond may experience disease progression or unacceptable toxicity during therapy, potentially leading to loss of eligibility for curative-intent interventions.^95^ In addition, neoadjuvant regimens may be associated with significant toxicity. Treatment-related adverse events may lead to deterioration in liver function, which is a critical determinant of treatment eligibility in HCC.^96^ Such complications may increase the risk of patient dropout from planned curative procedures or subsequent therapies, thereby negatively affecting overall treatment outcomes. As a result, careful patient selection and close monitoring are essential to minimize these risks. According to a recent large-scale study from China, although the administration of neoadjuvant immunotherapy with transarterial chemoembolization (TACE) and TKIs provided better response and survival rates compared to surgery alone without neoadjuvant therapy, it is critical to mention that the administration of the neoadjuvant therapy was associated with more frequent serious adverse events (almost in half of patients) and postoperative complications, such as post-resection liver failure, bile leakage and ascites.^97^ Furthermore, approximately half of the patients from the neoadjuvant group of this study experienced an event (death, disease progression or recurrence) while on treatment, a fact that should be carefully taken into consideration when assessing patients for the administration of such therapies. According to a recent meta-analysis, the overall rate of pathologic complete response (pCR) with different immunotherapy regimens in the neoadjuvant setting was 15%, with a major pathologic response (MPR) rate of 28%, leaving a big gap of approximately 70% for non-responders, that will either not achieve conversion or even worse experience progressive disease and therefore will then be totally excluded from radical treatments.^98^ Furthermore, according to the same study, single-agent immunotherapy is associated with a better safety profile than dual-agent immunotherapy or combined immunotherapy with TKIs in the neoadjuvant setting. Moreover, early exposure to immunotherapy in this context may limit subsequent treatment options in the event of progression, as resistance mechanisms may reduce the efficacy of later-line immunotherapeutic strategies.^99^ In such cases, treatment options may be restricted to TKIs, which are associated with comparatively modest response rates and survival outcomes.^100^

Another important consideration is the potential delay or deferral of established locoregional therapies. In patients with intermediate-stage disease, treatments such as TACE, transarterial radioembolization (TARE) and, more recently, stereotactic body radiation therapy (SBRT), have demonstrated clinical benefit and remain standard therapeutic options.^101^^,^^102^^,^^103^ The introduction of neoadjuvant systemic therapy may postpone the use of these modalities, potentially impacting disease control in selected patients.

## Current data from clinical trials on neoadjuvant therapies

Emerging evidence on neoadjuvant systemic therapies in HCC is primarily derived from early-phase clinical studies evaluating ICI-based approaches. The results from the most important clinical trials with ICIs in the neoadjuvant setting are shown in Table 2. Initial trials investigating anti-PD1 monotherapy, such as tislelizumab, have demonstrated that neoadjuvant treatment is feasible and can be administered without compromising the safety or timing of subsequent surgical resection.^104^ For instance, a recent phase II study of neoadjuvant nivolumab with 43 patients reported that neoadjuvant treatment was associated with pathological responses reaching approximately 26%.^105^ In another study with 21 patients, the anti-PD1 agent cemiplimab managed to lead to significant tumor necrosis in 20% of patients, with one-third of patients experiencing serious adverse events.^106^ The combination of immunotherapies has also been studied. More specifically, the combination of nivolumab with ipilimumab in the neoadjuvant setting in patients with intermediate and advanced HCC managed to downstage almost 50% of patients, leading to a curative resection, with 4-year OS and RFS rates reaching up to 60% and 44%, respectively, while almost 20% of patients achieved MPR.^107^ Double neoadjuvant immunotherapy with anti-PD1 and anti-LAG3 molecules has also shown promising preliminary results according to a recent study with 30 patients.^108^ Anti-VEGF monoclonal antibodies have also been studied. In a neoadjuvant HCC study with 30 patients, the combination of sintilimab with a bevacizumab biosimilar achieved ORRs of 26.7% and disease control rates of 90%, leading to liver resection in more than half of patients with manageable efficacy.^109^ These findings suggest that even short courses of preoperative immunotherapy may induce meaningful immune activation and tumor regression. However, the clinical significance of these responses remains uncertain, given the small sample sizes and heterogeneity in patient selection.

**Table 2:** Published results from major neoadjuvant immunotherapy studies in HCC

Study (Ref)	NCT	Phase	N	Treatment regimen	Primary Endpoint(s)	Key results
Chen et al.^104^	NCT04425226 (completed)	phase II	11	tislelizumab	1-year DFS	MPR 18% ORR 18% pCR 9%
Nahon et al.^105^	NCT03630640 (completed)	phase II	43	nivolumab	1-year LRFS	1-year LRFS 70% 2-year OS 74%
Marron et al.^106^	NCT03916627 (completed)	phase II	21	cemiplimab	significant tumor necrosis (>70%)	significant tumor necrosis 20% ORR 15%
Lin et al.^107^	NCT03222076 (completed)	phase II	43	nivolumab + ipilimumab	downstaging rate, pathological response	downstaging 55% MPR 18% 4-year OS 60% 4-year RFS 44%
Li et al.^108^	NCT04658147 (interim analysis)	phase II	30	nivolumab ± relatlimab	feasibility for surgery	resection 73.3-86.7% MPR 20–26.7% ORR 13–20% 2-year DFS 73–89%
Sun et al.^109^	NCT04072679 (completed)	phase II	30	sintilimab + bevacizumab biosimilar	EFS safety	ORR 26.7% DCR 90% resection 56% 1-year EFS 60%
Sun et al.^110^	NCT05389527 (interim analysis)	phase II	43	pembrolizumab + lenvatinib	MPR	resection 93% MPR 38.5% pCR 7.7%
Bai et al.^111^	NCT04639180 (interim analysis)	phase II/III	24	camrelizumab + apatinib	1-year EFS	surgery 75% ORR 62.5% MPR 25%
Ho et al.^75^	NCT03299946 (completed)	phase Ib/II	15	cabozantinib + nivolumab	conversion to resectability	downstaging 80% MPR 42%
Shi et al.^112^	NCT03804281 (completed)	phase Ib/II	18	toripalimab ± lenvatinib	pathological response	MPR 20% pCR 0%

NCT: national clinical trial; DFS: disease-free survival; MPR: major pathological response; ORR: objective response rate; pCR: pathological complete response; LRFS: local recurrence-free survival; OS: overall survival; RFS: recurrence-free survival; EFS: evente-free survival; DCR: disease control rate.

Building upon these initial results, combination strategies incorporating ICIs with anti-angiogenic agents or TKIs have been increasingly explored. Lenvatinib combined with pembrolizumab in a study of 43 patients have demonstrated encouraging ORRs and high rates of tumor downstaging with MPRs reaching 37.8%, though the results of this study are only preliminary.^110^ In some cases, these combinations have enabled conversion to resectability in patients initially deemed unsuitable for curative-intent treatment. Similar results have also been reported from some other completed or ongoing studies, although with limited numbers of patients.^75^^,^^104^^,^^111^^,^^112^

An additional area of active investigation involves the integration of neoadjuvant systemic therapy with locoregional treatments. The rationale for combining immunotherapy with locoregional treatments such as TACE has already been proved to be beneficial in three different randomized clinical trials, which recruited patients with HCC in the intermediate stage.^113^^,^^114^^,^^115^ In this direction, the combination could also be used as a neoadjuvant treatment in patients who exceed common resection criteria, in order to achieve a radical treatment. As presented in Table 3, several clinical trials that have not yet reported efficacy results are currently evaluating combinations of ICIs with TACE, TARE or SBRT in the neoadjuvant setting, based on the hypothesis that locoregional tumor necrosis may enhance antigen release, converting a “cold” tumor to “hot” and synergize with immunotherapy.^116^ On this basis, a wide variety of clinical studies that assess the efficacy of neoadjuvant locoregional therapies with or without the concurrent use of immunotherapy have been developed.

**Table 3:** Neoadjuvant immunotherapy clinical trials without published efficacy results

NCT number	Study Title	Interventions	Primary endpoints	Start year	Completion year
NCT07522411	sequential FOLFOX-HAIC-TACE Plus Sintilimab-bevacizumab neoadjuvant therapy versus direct resection in resectable high-risk recurrence hepatocellular carcinoma	FOLFOX + immunotherapy + targeted therapy + cTACE	RFS	2026	2028
NCT07154082	apatinib mesylate and camrelizumab combined with TACE as neoadjuvant therapy for hepatocellular carcinoma	TACE + targeted or immunotherapy	DFS	2025	2028
NCT06045975	apatinib mesylate and camrelizumab in neoadjuvant and adjuvant setting in patients with HCC treated by percutaneous ablation procedure	durvalumab/tremelimumab (neoadjuvant) durvalumab (adjuvant)	Local RFS	2024	2028
NCT06492408	neoadjuvant intra-tumor double immunotherapy for hepatocellular carcinoma	ipilimumab, pembrolizumab, durvalumab, idarubicin, bevacizumab	pCR rate	2024	2033
NCT07075120	ipilimumab and nivolumab with SBRT in locally advanced hepatocellular cancer	ipilimumab nivolumab stereotactic body radiotherapy (SBRT)	feasibility of treatment regimen	2026	2027
NCT06954116	iparomlimab and tuvonralimab as neoadjuvant therapy for resectable hepatocellular carcinoma with high risk of recurrence	iparomlimab tuvonralimab (QL1706) partial hepatectomy	RFS	2025	2029
NCT07489976	IIT2025-03-YANG-LIFT-HCC	STRIDE Regimen LRT (TACE/TARE/RFA)	proportion of participants downstaged from beyond UCSF criteria to within Milan criteria.	2026	2030
NCT06311916	efficacy and safety of neoadjuvant therapy in patients with resectable HCC screened by a multimodal deep learning model	HAIC + tirelizumab + lenvatinib + liver resection	DFS	2024	2028
NCT07009470	a multicenter, prospective study of perioperative finotonlimab combined with Bevacizumab in resectable hepatocellular carcinoma patients with high-risk factors for recurrence	finotonlimab (anti-PD-1) anbeizhu (a bevacizumab biosimilar) TACE	2-year DFS	2025	2028
NCT05908786	a study evaluating the efficacy and safety of neoadjuvant immunotherapy combinations in patients with surgically resectable hepatocellular carcinoma	atezolizumab bevacizumab tiragolumab tobemstomig	MPR	2023	2025 no published results
NCT04721132	atezolizumab and bevacizumab before surgery for the treatment of resectable liver cancer	atezolizumab bevacizumab	MPR safety	2021	2027

NCT: national clinical trial; RFS: recurrence-free survival; cTACE: conventional transarterial chemoambolization; HAIC: hepatic artery infusion chemotherapy; TACE: transarterial chemoembolization; DFS: disease-free survival; HCC: hepatocellular carcinoma; pCR: pathological complete response; SBRT: stereotactic body radiation therapy; STRIDE: single trmelimumab Regular dose Durvalumab; LRT: locoregional therapy; TARE: transarterial radioembolization; RFA: radiofrequency ablation; UCSF: University of California San Fransisco; MPR: major pathological response. Studies included in this table have not yet reported efficacy or survival results. Final analyses are awaited.

Despite all the aforementioned encouraging findings, the current body of evidence supporting neoadjuvant therapy in HCC remains limited and heterogeneous, and as a result, neoadjuvant therapies have not yet been adopted by most HCC guidelines.^15^ Most available studies are phase I–II, single-arm trials with small patient populations, short follow-up durations and variable endpoints. Furthermore, there is no standardized definition of pathological response in HCC, and surrogate endpoints such as MPR or pCR have not yet been validated as predictors of long-term outcomes and there are major discrepancies between their definitions across different studies.^117^

## Perioperative therapy: a promising ally

Following the evaluation of both adjuvant and neoadjuvant strategies in HCC, increasing attention has been directed toward their combination as perioperative systemic therapy.^118^ This approach is based on a compelling biological rationale, whereby neoadjuvant immunotherapy administered in the presence of an intact tumor enables robust immune priming through exposure to a wide variety of tumor-associated neoantigens. Subsequently, the continuation of systemic therapy in the adjuvant setting aims not only to sustain but also to further amplify the anti-tumor immune response, thereby achieving long-term disease control.^119^

It is important to emphasize that hepatocarcinogenesis is driven by a characteristic triad consisting of the pre-oncogenic dynamics of the cirrhotic liver, an immunosuppressive tumor microenvironment and the presence of malignant hepatocyte clones.^120^ Within this complex biological framework, the need for both neoadjuvant and adjuvant therapeutic strategies becomes increasingly apparent. Adjuvant therapy alone may result in suboptimal immune activation, as it is administered in the context of minimal residual disease with limited antigenic stimulation.^121^ Conversely, neoadjuvant treatment followed by surgery without continuation of systemic therapy may fail to sustain the anti-tumor immune response and may even predispose to tumor progression. This phenomenon is partly attributed to postoperative liver regeneration, which is associated with the release of pro-inflammatory and pro-angiogenic cytokines that may promote the growth of previously undetected microscopic disease and contribute to early recurrence.^122^ These observations challenge the traditional paradigm that early recurrence is primarily driven by residual disease and late recurrence by *de novo* carcinogenesis, as emerging data suggest a more complex overlap between these two mechanisms.^123^

In this context, the continuation of neoadjuvant therapy into the postoperative period as part of a perioperative strategy may represent a critical advantage. Unlike neoadjuvant therapy alone, which primarily enhances immune priming, the combined neoadjuvant-adjuvant approach allows for both the induction and maintenance of an effective anti-tumor immune response.^124^ This sustained immune surveillance may be essential for controlling microscopic residual disease and mitigating the protumorigenic effects of postoperative liver regeneration.^125^ Therefore, perioperative strategies may offer a more comprehensive therapeutic framework by targeting both macroscopic tumor burden prior to surgery and microscopic disease following curative-intent treatment, ultimately reducing recurrence risk and improving long-term outcomes.

The concept of perioperative immunotherapy in HCC was first supported by a randomized, open-label phase II trial evaluating perioperative nivolumab alone or in combination with ipilimumab in patients with resectable HCC.^126^ In this study, patients received short-course neoadjuvant immunotherapy followed by surgical resection and prolonged adjuvant immunotherapy for up to two years. The primary endpoint of safety and tolerability was successfully met, as no patient experienced a delay in surgery due to treatment-related adverse events, despite a higher incidence of grade 3–4 toxicities with the combination regimen compared with nivolumab monotherapy (43% vs. 23%). MPR was observed in approximately one-third of resected patients, irrespective of treatment arm, and patients achieving an MPR demonstrated durable recurrence-free outcomes, supporting pathological response as a potential surrogate marker of long-term benefit. Although limited by its small sample size and exploratory design in terms of efficacy, this landmark study established the feasibility and biological rationale of perioperative immune checkpoint inhibition in resectable HCC and laid the foundation for the development of larger phase III prospective studies that followed.

The CARES-009 trial represents the first large-scale randomized controlled phase III study to evaluate this perioperative concept in HCC.^127^ This trial investigated the perioperative combination of camrelizumab with rivoceranib, compared with surgery alone. The study population included patients across early, intermediate and selected advanced stages of HCC, provided there was no portal vein trunk invasion or extrahepatic disease. The results demonstrated a significant improvement in event-free survival (EFS) in the active arm compared to surgery alone (42.1 months versus 19.4 months, respectively). The trial also reported encouraging pathological responses, with approximately 35% of patients achieving MPR and 3.4% achieving pCR. Combined treatment discontinuation rates in the pre- and postoperative setting were 16% in total, with only 13% of patients experiencing serious treatment-related adverse events. These findings further support the concept of effective immune priming in the neoadjuvant setting with manageable safety. However, the study population consisted almost exclusively of patients of Asian origin, raising concerns regarding the generalizability of these findings to non-Asian populations.

Additional evidence supporting the role of perioperative approaches is provided by the TALENTop study, which evaluated the combination of atezolizumab plus bevacizumab with or without surgical resection in patients with advanced HCC and macrovascular invasion.^128^ In this study, all patients initially received first-line systemic therapy with atezolizumab and bevacizumab. Patients demonstrating stable disease or objective response and deemed resectable were allocated to a surgery arm, followed by continuation of systemic therapy for at least one year, while the remaining patients continued systemic therapy alone. The study introduced a novel endpoint, which was time to treatment failure, and demonstrated superior outcomes in the group receiving combined immunotherapy and surgery compared to immunotherapy alone. Although OS data were immature at the time of initial analysis, a clear trend toward improved survival was observed in the surgical cohort. Importantly, the findings from the TALENTop study suggest that even patients with more advanced disease may achieve meaningful oncologic benefit, including long-term disease control or even cure, when treated within a multimodal perioperative framework.

Taken together, these emerging data from large-scale clinical trials on the biologic basis of perioperative treatment support the concept that perioperative systemic therapy may represent a pivotal evolution in the management of HCC, though data are still immature and, subsequently, perioperative treatment should be interpreted more as an investigational approach rather than a standard-of-care treatment. By combining the immunological advantages of neoadjuvant treatment with the sustained disease control offered by adjuvant therapy, this approach has the potential to overcome many of the limitations observed with either strategy alone. While further validation in larger, randomized trials and more diverse populations is required, perioperative immunotherapy has the potential to redefine treatment paradigms in HCC, expanding the boundaries of curative-intent therapy and offering new hope for patients with either early or intermediate stage HCC and even carefully selected patients in advanced stages of the disease.^129^

## Future perspectives

**Figure 3:**
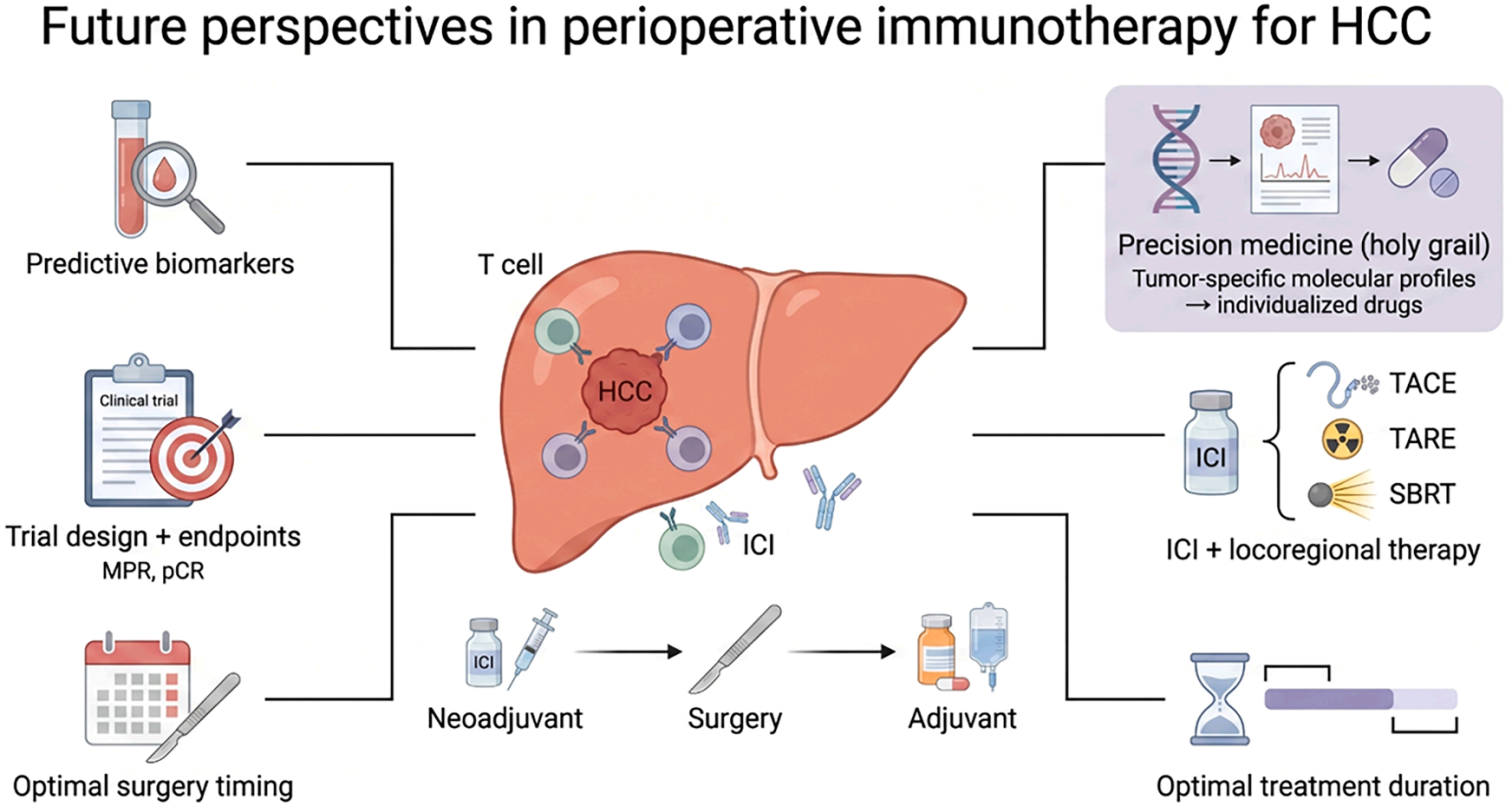
Future perspectives in perioperative immunotherapy for hepatocellular carcinoma (HCC) This schematic illustrates key future directions in perioperative immunotherapy for HCC. It highlights the integration of immune checkpoint inhibitors (ICIs) across the treatment continuum, from neoadjuvant to adjuvant settings, aiming to enhance anti-tumor immunity and reduce recurrence. Key areas in the development of this strategy include the identification of predictive biomarkers, optimization of clinical trial endpoints such as major pathological response (MPR) and pathological complete response (pCR), and determination of optimal surgical timing and treatment duration. The figure also emphasizes the potential synergy between ICIs and locoregional therapies, including transarterial chemoembolization (TACE), transarterial radioembolization (TARE) and stereotactic body radiation therapy (SBRT), which may promote immune activation. Ultimately, these strategies may facilitate the transition toward precision medicine through individualized, biology-driven therapeutic approaches, mainly through unraveling the tumor microenvironment.

The key future directions in the perioperative setting of HCC immunotherapy are presented in Figure 3. The evolving landscape of systemic therapy in HCC is increasingly shifting toward earlier stages of disease, with perioperative strategies emerging as a promising avenue to improve long-term outcomes.^130^

A major priority for future research is the identification of reliable predictive biomarkers that can guide patient selection. At present, there are no widely validated biomarkers capable of accurately predicting response to ICIs in HCC.^131^ Conventional markers such as PD-L1 expression and tumor mutational burden have shown limited utility in this disease.^132^ Emerging technologies, such as liquid biopsy-based approaches, hold promise for the detection of minimal residual disease and real-time monitoring of treatment response.^133^ However, their high cost is an important drawback; their clinical applicability remains investigational and further studies are required to establish their correlation with intrahepatic tumor biology and clinical outcomes.

Another key challenge lies in optimizing clinical trial design in the perioperative setting. Future studies should aim to standardize definitions of pathological response, including MPR and pCR, and to validate their association with long-term endpoints such as RFS and OS. In addition, the heterogeneity of HCC populations with respect to tumor burden, histological features, underlying liver disease severity, etiology and comorbidities necessitates more refined stratification strategies in order to identify subgroups most likely to benefit from perioperative interventions. Furthermore, the presence of TLSs in the tumor is an essential emerging biomarker that could predict responses not only in the adjuvant, but also in the neoadjuvant immunotherapy setting and could guide patient selection for clinical trials in the future, leading to a more individualized approach through precision medicine.

The optimal sequencing and duration of systemic therapy also remain to be defined. Questions regarding the ideal timing of surgery following neoadjuvant treatment, the necessity, timing and duration of adjuvant therapy after resection and the management of patients who achieve significant pathological responses are currently unresolved.^134^ Furthermore, the safety of perioperative immunotherapy, particularly in patients undergoing liver transplantation, represents an area of active investigation, given the potential risk of immune-mediated graft rejection.^135^ Importantly, the combination of systemic therapies with locoregional treatments is likely to play a central role in future treatment algorithms, taking into account the higher ORRs and the significantly longer RFS observed in all currently available randomized clinical trials with the combination compared to TACE alone in patients with BCLC-B staged HCC. The combination of ICIs with TACE, TARE and SBRT may enhance tumor antigen release and immune activation, thereby converting non-immune into immune “friendly” tumors that are more responsive to immunotherapy.^136^

Ultimately, the “holy grail” in the adjuvant, neoadjuvant and perioperative immunotherapy setting would be the identification of tumor-specific molecular profiles for each individual case of HCC, paving the way toward precision medicine.^137^ In this context, it is crucial to consider the independent carcinogenic potential of the cirrhotic liver, which may give rise to multiple distinct clonal tumor populations, as well as the unique mutational landscape of each tumor and the complex biological processes occurring within the tumor microenvironment.^138^ The latter can be characterized through the evaluation of tumor-associated immune cell populations and their interactions, including tumor-associated neutrophils and macrophages, natural killer cells, intratumoral effector T cells, dendritic cells, as well as regulatory T cells and myeloid-derived suppressor cells.^139^ The dynamic interplay among these components may provide critical insights into tumor behavior and immune responsiveness, ultimately enabling a more individualized therapeutic approach. Such strategies could be particularly impactful not only in advanced HCC but also in the perioperative setting, where tailored modulation of the tumor microenvironment may enhance treatment responses and translate into improved long-term survival outcomes.

Taken together, future efforts in this field should focus on refining patient selection, optimizing treatment sequencing and integrating translational biomarkers into clinical practice. The apparent discrepancy between the limited efficacy of adjuvant therapies and the emerging promise of neoadjuvant approaches underscores the importance of tumor-immune system interactions in HCC. Adjuvant therapies, before being abandoned, should be tested in a highly selected patient population that will have high HCC recurrence risk after resection or ablation, as well as biological markers within the tumor microenvironment that could predict response to ICIs, such as the presence of TLSs or the absence of CD163^+^ tumor-associated macrophages or vascular cancer-associated fibroblasts, a higher Treg/Teff ratio and pro-tumor supportive cellular interactions.^140^ Perioperative immunotherapy may ultimately represent a paradigm shift in the management of early and intermediate-stage HCC, with the potential to expand the boundaries of curative-intent treatment and improve long-term patient outcomes even in more advanced HCC stages. At its core, the central question moving forward is whether tumor biology will permit cure through liver resection alone, or whether biological modulation is required to achieve durable disease control. Given the persistently high rates of recurrence following surgery, it is increasingly evident that resection alone may be insufficient in a substantial proportion of patients. Therefore, the future of HCC management may lie not only in removing the tumor, but in actively reshaping its biological behavior prior to the development of recurrence.

## Conclusions

HCC is associated with high recurrence rates despite curative-intent treatments, highlighting a critical unmet need for effective perioperative strategies. While adjuvant therapies have shown limited and inconsistent benefit, emerging evidence suggests that neoadjuvant approaches may offer greater potential, largely due to enhanced immune priming in the presence of an intact tumor. Building on this concept, perioperative strategies that combine neoadjuvant and adjuvant therapy may provide a more comprehensive approach to disease control, targeting both macroscopic and microscopic disease. Early clinical studies have demonstrated encouraging efficacy and acceptable safety profiles, supporting the biological rationale for this therapeutic strategy. However, the currently available evidence is largely derived from early-phase clinical trials and mature survival data from randomized phase III studies remain limited. Future progress will depend on optimizing patient selection through biomarker-driven approaches, refining treatment sequencing and validating pathological and molecular predictors of response. Overall, perioperative immunotherapy represents a promising investigational strategy for selected patients with HCC, but its role in routine clinical practice will require confirmation through adequately powered randomized trials with mature long-term survival outcomes.

## Acknowledgments

The authors of this review have nothing to acknowledge.

## Declaration of interests

The authors declare no competing interests.
